# Uptake of intermittent preventive treatment with sulphadoxine-pyrimethamine for malaria during pregnancy and pregnancy outcomes: a cross-sectional study in Geita district, North-Western Tanzania

**DOI:** 10.1186/1475-2875-13-455

**Published:** 2014-11-24

**Authors:** Filbert J Mpogoro, Dismas Matovelo, Aliyah Dosani, Sospatro Ngallaba, Moshi Mugono, Humphrey D Mazigo

**Affiliations:** School of Public Health, Catholic University of Health and Allied Sciences- Bugando, PO Box 1464, Mwanza, Tanzania; Department of Obstetrics and Gynaecology, Catholic University of Health and Allied Sciences- Bugando, PO Box 1464, Mwanza, Tanzania; School of Nursing and Midwifery, Mount Royal University, 4825 Mount Royal Gate SW, Calgary, AB T3E 6 K6 Canada; Department of Parasitology and Entomology, Catholic University of Health and Allied Sciences- Bugando, PO Box 1464, Mwanza, Tanzania

**Keywords:** Pregnancy, Intermittent preventive treatment, Sulphadoxine-pyrimethamine placental malaria, Pre-term delivery, Low birth weight, Tanzania

## Abstract

**Background:**

Malaria infection during pregnancy is associated with adverse outcomes in sub-Saharan Africa (SSA). For this reason, the World Health Organization currently recommends intermittent preventive treatment of malaria in pregnancy (IPTp) with sulphadoxine-pyrimethamine (SP) at each scheduled antenatal care (ANC) visit. In Tanzania, the revised IPTp policy was adopted in 2013 but the level of uptake and its association with pregnancy outcomes remains unknown.

**Methods:**

A cross-sectional study was conducted among singleton pregnant women who delivered in two selected health facilities of Geita district, northwestern Tanzania. Self-reported uptake of SP was verified using the ANC card and was recorded. Placental and peripheral blood was collected for diagnosis of malaria by microscopy and rapid diagnostic tests (RDTs). Gestational age was estimated based on last menstrual period or Ballard score. Infant birth weights were recorded within 24 hours of delivery.

**Results:**

Of 431 participants, 167 (38.75%), 134 (31.09%), 104 (24.23%), and 26 (6.03%) reported taking none, one, two, and ≥ three doses of SP during pregnancy, respectively. The uptake of ≥ three doses of IPTp-SP among delivering women at Geita hospital and Katoro health centre was 9.06% and 1.2%, respectively. The overall prevalence of malaria in pregnancy by RDT, peripheral and placental smears was 19.5%, 29.7% and 37.6% respectively. The prevalence of placental parasitaemia was higher for women who delivered at Katoro Health Centre (41.57%) than those who delivered at Geita hospital (35.09%). The uptake of ≥ three doses of SP was associated with reduced odds of having placental malaria (adjusted odds ratio (AOR) = 0.31, *p =* 0.039) compared to < three doses. Women with placental parasitaemia were five times more likely to have delivered pre-term (AOR = 4.67, *p =* 0.002) and had lower mean birth weight infants than their uninfected counterparts (mean difference = 82 g, *p =* 0.039).

**Conclusions:**

The uptake of ≥ three doses of IPTp-SP is low in the present study area. Placental parasitaemia is prevalent and is associated with adverse birth outcomes. Receipt of ≥ three doses of IPTp-SP reduced the odds of placental parasitaemia. Thus, increased efforts towards scale-up and continuous evaluation of IPTp-SP efficacy is recommended.

## Background

Of the 125 million pregnant women who are at risk of *Plasmodium falciparum* infection each year, 30 million are from SSA [1]. *Plasmodium falciparum* malaria infection during pregnancy presents significant risks for the pregnant woman, the developing foetus and the new-born infant. The negative consequences associated with malaria in pregnancy include: severe malaria, severe anaemia, pre-term delivery, maternal death, and placental malaria [2–4]. Placental malaria is linked to intra-uterine growth restriction, stillbirth, and delivery of low birth weight (LBW) infants [5, 6]. Pre-term delivery and LBW are the risk factors for neonatal and infant deaths [7]. In Africa where malaria is endemic, malaria-causing LBW kills between 62,000 and 363,000 new-borns every year [8, 9]. In Tanzania, 93% of the population is at risk of malaria infection. *Plasmodium falciparum* malaria is responsible for up to 125,000 deaths in Tanzania, of which, 70,000-80,000 deaths occur in children who are under five years old [10]. The disease accounts for at least one fifth of all maternal deaths in Tanzania [11]. To prevent the effects of *P. falciparum* malaria during pregnancy, the World Health Organization (WHO) currently recommends intermittent preventive treatment of malaria in pregnancy (IPTp) using sulphadoxine-pyrimethamine (SP) at each scheduled antenatal care (ANC) visit after the first trimester [12]. In SSA, the effectiveness of IPTp-SP in preventing malaria-related adverse pregnancy outcomes is well established [13–15]. Despite the widespread adoption of IPTp in malaria-endemic countries in Africa, the coverage of recommended doses of IPTp-SP remains elusive [16]. In 2012, the median coverage of at least one, two and three doses of SP during pregnancy in SSA was 64% (range 25-85%), 38% (range 10-64%), and 23% (range 2-44%), respectively [17]. In Tanzania, the coverage of at least one and two doses as per Tanzania HIV/AIDS Malaria Indictor Survey 2011/12 was 60% and 32%, respectively [18]. In addition, the widespread SP resistance caused by *P. falciparum* dihydrofolate reductase/dihydropteroate synthase *(pfdhfr/pfdhps*) quintuple or sextuple mutations [19, 20] is likely to affect the efficacy of SP in East Africa, including Tanzania. However, a recent systematic review and meta-analysis showed beneficial effects of three or more doses of SP during pregnancy in prevention of malaria-related adverse outcomes, even in areas with moderate to high SP resistance [21]. The new IPTp regimen has recently been implemented in Tanzania; thus the level of uptake and its association with pregnancy outcomes remains unknown in various regions of the country. The purpose of the present study was to determine the uptake of SP among pregnant women based on the revised recommendations, and to evaluate its association with adverse pregnancy outcomes, including placental malaria, pre-term delivery and LBW.

## Methods

### Study sites, design, participants and selection

This analytical cross-sectional study was conducted from May to June 2014 in delivery units at Geita district hospital and Katoro health centre, north-western Tanzania. Geita hospital is located within the capital city of Geita region (urban) while Katoro rural health centre is located 45 kilometre from Geita city (peri-urban). The inhabitants of Geita district are predominantly subsistence farmers and belong to the Sukuma and Zinza ethnic groups. Their main activities are farming, livestock keeping, small business, fishing, and mining. Malaria is highly endemic in this area with highest transmission occurring between March and June. Lake Zone has more than 80% prevalence of the *pfdhfr/pfdhps* quintuple mutant of IRNGE haplotype associated with SP resistance [22]. Pregnant women aged 15–49 years delivering at Geita district hospital or Katoro health centre were assessed for enrolment. Women were enrolled in our study if they fulfilled the following inclusion criteria: 1) singleton pregnancy; 2) seronegative for HIV; 3) absence of reported antimalarial treatment other than SP in the previous one month; and, 4) consented to be included in the study. Convenience sampling was used to recruit pregnant women who met the inclusion criteria and consented to participate in the study. Study participants were enrolled until the required sample size was reached.

### Sample size

The two-proportion sample size formula without the correction factor by Fleiss [23] at 90% power level and 5% level of significance was used to estimate the required sample size. A minimum sample size of 426 women was calculated by considering the previous study that reported the proportion of women with placental malaria among ≥ three doses of SP group of 8% and that among < three doses of SP group of 17% [13]. A total of 431 delivering women in selected health facilities were recruited into the study.

### Data collection

#### Demographic and obstetric information

Information was collected using an interviewer-administered structured questionnaire. The questionnaire collected socio-demographic information such as maternal age, marital status, education, and occupation. Also, obstetric history on parity, timing and frequency of ANC visits were collected. ANC cards were examined and information on the use of SP during pregnancy, number of doses taken, gestational age at first and subsequent SP doses, and the time lapse from the last dose to delivery were recorded. The ANC card also provided information on gestational age at first ANC clinic visit and frequency of ANC clinic attendance.

Birth weights of new-borns were measured within 24 hours of birth using SECA weighing scales (Vogel & Halke Gmbh & Co, Hamburg, Germany). Gestational age was estimated based on last normal menstrual period, and if uncertain and no early ultrasound scans available, the new Ballard score maturational assessment [24] was done within 24 hours of delivery by trained midwives.

#### Blood sample collection, processing and examination of malaria parasites and antigens

Malaria infection was screened in both placental and maternal peripheral blood samples by microscopy and rapid diagnostic tests (RDTs). Two mL of maternal peripheral venous blood were collected immediately after delivery, from which a thick blood smear was prepared and malaria RDT was done. Placental blood was collected using the procedures described elsewhere [25], for thick smear preparation. Both peripheral and placental blood samples were collected into the vacuum tubes containing ethylene diamine tetra acetic acid (EDTA) anticoagulants and smears were prepared within one hour after collection.

Maternal blood samples were evaluated by histidine rich protein II (HRP II) and parasite lactate dehydrogenase (*p*LDH) antigen-based malaria RDTs (Standard Diagnostic Inc, South Korea). RDTs were performed according to the manufacturer’s instructions and examined after 15 min following addition of four drops of the wash buffer.

Thick smears prepared from peripheral and placental blood samples were fixed with absolute ethanol and stained with 10% Giemsa for 30 min [26]. Slides were examined by two independent laboratory technicians using light microscopy x 100 objectives under oil immersion for the presence of any malaria parasites stages. To minimize observation bias, the laboratory technicians reading the placental and peripheral smears were blinded to results of RDTs. Discordant results were given a third reading by a microbiologist blinded to the results of the first two readings, the result of which was considered final. A thick film was considered positive when any stage of malaria parasites were detected. At least 100 high power fields were examined before a thick smear was declared negative. Malaria parasites were counted against 500 leucocytes and parasite densities expressed per μl blood, assuming a white blood cell count of 8,000/μl blood. The parasite density (parasite/μL of blood) was then calculated as follows: Parasite density = (number of parasites counted/leukocytes counted) × white blood cell count/μL blood [27].

### Definitions

IPTp-SP was defined as provision of treatment doses of SP to asymptomatic pregnant women living in malaria-endemic regions, regardless of malaria parasitaemia status. In areas of stable malaria transmission, WHO currently recommends IPTp-SP for all pregnant women at each scheduled ANC visit provided that each dose is taken four weeks apart from the other and continuing up to the time of delivery. Paucigravidae was defined as women in their first and second pregnancies and multigravidae referred to women with three or more pregnancies. Placental malaria was defined as direct detection of asexual stage of malaria parasites in placental thick smears regardless of *Plasmodium* species. Birth weights below 2,500 g measured within 24 hours of birth was regarded as LBW. Pre-term delivery was defined as delivery of an infant before 37 weeks gestational age.

### Data analysis

Data collected were double-entered and cleaned using Epi-Data version 3.1 (CDC, Atlanta, GA, USA), and then exported to STATA version 12.0 (Stata Corp, College Station, TX, USA) for analysis. The primary exposure variable of interest was IPTp-SP usage, categorized into < three doses *versus* ≥ three doses of IPTp. Data were summarized in the form of proportions and frequency tables for categorical variables. Means with their respective standard deviations were used to summarize continuous variables.

Bivariate logistic regression analyses were performed to determine the presence or absence of association between SP usage and placental malaria. All explanatory variables with *p* <0.20 in the bivariate analysis, were included in the multivariable analysis to further examine the association between IPTp-SP use and placental malaria. Secondary analyses were done to determine association between placental malaria and birth outcomes (birth weight and pre-term delivery) using logistic and linear regression analyses. Adjusted odds ratio (AOR), regression coefficients and 95% confidence intervals were reported. The level of significance was considered at 5%.

### Ethical considerations

Written informed consent for study participation was obtained from all participants aged 18 years and above. For women aged below 18 years, informed assent was obtained and their husbands, mothers or mother in-laws provided informed consents. The study was approved by the Joint Catholic University of Health and Allied Sciences (CUHAS)/Bugando Medical Centre (BMC) Research Ethical Committee (Certificate No. CREC/016/2014) and local authorities. Women found to have malaria at delivery were treated with the anti-malarial treatment protocol in line with national guidelines.

## Results

### Study participant characteristics

A total of 431 women were enrolled out of 1,701 total deliveries in Geita district hospital and Katoro rural health centre from May-June 2014. The demographic characteristics of the 431 women with singleton deliveries who participated in the present study are shown in Table 1. The participants were aged between 15–45 years, and the majority (77.49%) were aged above 20 years. Nearly two-thirds (61.48%) of the women delivered at Geita district hospital. Of 431 delivering women, one-third had previously visited at least four or more antenatal care visits. Of all the study participants, 227 (52.67%) were in their first and second pregnancies. Majority (82.83%) of women were married, 85.53% had primary level or no formal education and 85.85% were peasants and/or keeping livestock.

**Table 1:** **Characteristics of 431 women with singleton deliveries at Geita district hospital and Katoro health centre**

Variable	No (%)
**Maternal age (years)**	
<20	97 (22.51)
≥20	334 (77.49)
**Marital status**	
Single	74 (17.17)
Married	357 (82.83)
**Health facility**	
Geita Hospital	265 (61.48)
Katoro HC	166 (38.52)
**Gravidity**	
Paucigravidae (G1/G2)	227 (52.67)
Multigravidae (G3+)	204 (47.33)
**Level of education**	
None/Primary	360 (83.53)
Secondary/College	71 (16.47)
**Occupation**	
Peasants/livestock keeping	370 (85.85)
Employed/business	61 (14.15)
**ANC visits**	
<4 visits	276 (64.04)
≥4 visits	155 (35.96)

ANC = antenatal care, SP = sulphadoxine-pyrimethamine, G1 = primigravidae, G2 = secundigravidae, G3+ = gravida 3 and above, HC = health centre, IPTp-SP = intermittent preventive treatment of malaria during pregnancy with SP.

### Uptake of IPTp-SP

Of 431 women delivering at selected health facilities, 26 (6.03%) received three or more doses of SP, 104 (24.13%) received two doses, 134 (31.09%) received one dose and 167 (38.74%) received no SP at all. Of 265 women who delivered at Geita hospital, 38 (14.34%) did not receive any dose of SP, 109 (41.13%) received one dose, 94 (35.47%) received two doses and only 24 (9.06%) took three or more doses of SP during pregnancy. Among 166 delivering women at Katoro health centre, 129 (77.71%) did not receive any dose, 25 (15.06%) received one dose, 10 (6.02%) received two doses and only 2 (1.2%) received the recommended three or more doses of IPTp-SP. On bivariate logistic regression analysis (Table 2), the uptake of ≥3 doses of SP during pregnancy was significantly associated with ≥ four ANC visits attended by the women (OR = 4.41, 95% CI, 1.87-10.42, *p* = 0.001), higher level of education (OR = 4.22, 95% CI, 1.85-9.62, *p* = 0.001), area of residence in urban setting, (OR = 8.20, 95% CI, 1.90-34.48, *p* = 0.005), and being employed or doing business (OR = 4.35, 95% CI, 1.85-10.10, *p* = 0.001). There was no significant difference in the uptake of SP during pregnancy between age groups, marital status and gravidity. In multivariable analysis (Table 2), women delivering at Geita hospital (AOR = 8.33, *p* = 0.007) and attendance of four or more ANC visits (AOR = 4.35, *p* = 0.001) were associated with uptake of ≥3 doses of IPTp-SP.

**Table 2:** **Factors associated with uptake of three or more doses of IPTp-SP among delivering women**

Variable	IPTp-SP uptake		COR (95% CI)	P-value	AOR (95% CI)	P-value
	N	≥3 doses, n (%)				
**Maternal age**						
<20 years	97	3 (3.09)	Reference		Reference	
≥20 years	334	23 (6.89)	2.33 (0.68-7.87)	0.179	2.33 (0.63-9.09)	0.203
**Marital status**						
Single	74	7 (9.49)	1.86 (0.75-4.60)	0.180	2.37 (0.84-6.67)	0.103
Married	357	19 (5.32)	Reference		Reference	
**Health facility**						
Katoro HC	166	2 (1.20)	Reference		Reference	
Geita hospital	265	24 (9.06)	8.20 (1.90-34.48)	**0.005**	8.33 (1.79-33.33)	**0.007**
**Level of education**						
None/Primary	360	15 (4.17)	Reference		Reference	
Secondary/Higher	71	11 (15.49)	4.22 (1.85-9.62)	**0.001**	1.69 (0.66-4.35)	0.275
**Occupation**						
Peasants	370	16 (4.32)	Reference		Reference	
Employed/business	61	10 (16.39)	4.35 (1.85-10.10)	**0.001**	1.85 (0.70-5.00)	0.214
**ANC visits**						
<4 visits	276	8 (2.90)	Reference		Reference	
≥4 visits	155	18 (11.61)	4.41 (1.87-10.42)	**0.001**	4.35 (1.79-11.11)	**0.001**

ANC: antenatal care; AOR: adjusted odds ratio; COR: crude odds ratio; IPTp-SP: intermittent preventive treatment of malaria in pregnancy with sulphadoxine-pyrimethamine; Significant p-values are presented in bold.

### Prevalence of malaria in pregnancy and birth outcomes

The overall prevalence of malaria in pregnancy based on malaria RDT was 19.49% (95% CI, 15.73-23.24), all of which occurred among women who received < three doses of SP. The prevalence of malaria in pregnancy by RDT among delivering women at Geita hospital and Katoro health centre was 13.58% and 28.92% respectively. The sensitivity of RDT in detection of specific parasite proteins was very low. Over half, 60.94% and 66.05% of women with peripheral and placental positive smears respectively had negative blood by RDT. Malaria *Plasmodium* species based on RDT were *P. falciparum,* 61.90% (n = 52), non-falciparum malaria parasites, 5.95% (n = 5) and mixed infections, 32.14% (n = 27).

The overall prevalence of peripheral malaria by microscopy was 29.70% (95% CI, 25.37-34.03). The prevalence of peripheral malaria by smear among delivering women at Geita hospital and Katoro health centre was 25.28% and 36.75% respectively. The overall geometric mean parasite density (GMPD) of peripheral parasitaemia was 167.75 (95% CI, 141.52-198.85) parasites/μL. Of 128 delivering women with peripheral parasitaemia, 73 (57.03%) had parasite density <100 parasites/μL.

The overall prevalence of placental malaria by microscopy was 37.60% (95% CI, 33.00-42.18). The prevalence of placental malaria by microscopy among delivering women at Geita hospital and Katoro health centre was 35.09% and 41.57% respectively. The overall GMPD (95% CI) of the placenta was 144.31 (126.25-164.95) parasites/μL. Among 162 delivering women with placental parasitaemia, 98 (60.49%) had parasite density <100 parasites/μL.

Of 431 delivering women at selected health facilities, 58 (13.46%) had pre-term deliveries. Of these, 54 (93.10%) had gestational age 34–36 weeks at delivery. The prevalence of pre-term delivery among women delivering at Geita hospital and Katoro health centre was 9.43% (n = 25) and 19.88% (n = 33) respectively. Of 431 women who delivered at selected health facilities, only 10 (2.32%) had low birth weight babies. Of 10 infants with LBW, 8 (80%) were born prematurely. The prevalence of LBW among delivering women at Geita hospital and Katoro health centre was 2.64% (n = 7) and 1.81% (n = 3) respectively.

### Factors influencing the placental parasite density

On bivariate analysis, young women <20 years (*p* = 0.049), delivering at Katoro rural health centre (*p* <0.001), having low level of education (*p* = 0.014) and being a peasant or keeping livestock (*p =* 0.034) had high placental parasite density. However, after adjusting for other covariates, women delivering at Katoro rural health centre (*p* <0.001) and non-use of any type of bed net (*p* = 0.030) remained independently associated with placental parasite density. Receiving less than three doses of IPTp-SP was a perfect predictor of >100 parasites/μl of placental samples (Table 3).

**Table 3:** **Analysis of factors associated with placental parasite density among delivering women**

Variable	Parasite density		Crude OR	P-value	Adjusted OR	P-value
	N	No. of PD >100/μl, (%)	(95% CI)		(95% CI)	
**Maternal age**						
<20 years	42	22 (52.38)	2.04 (1.00, 4.16)	**0.049**	1.98 (0.76, 5.17)	0.162
≥20 years	120	42 (35.00)	Reference		Reference	
**Health facility**						
Geita hospital	93	23 (24.73)	Reference		Reference	
Katoro HC	69	41 (59.42)	4.46 (2.27, 8.73)	**<0.001**	5.32 (2.45, 11.57)	**<0.001**
**Gravidity**						
Paucigravidae	94	43 (45.74)	1.89 (0.98, 3.63)	0.058	2.05 (0.85, 4.93)	0.110
Multigravidae	68	21 (30.88)	Reference		Reference	
**Level of education**						
None/Primary	140	61 (43.57)	4.89 (1.38, 17.29)	**0.014**	3.18 (0.80, 12.55)	0.099
Secondary/Higher	22	3 (13.64)	Reference		Reference	
**Occupation**						
Peasant/livestock	143	61 (42.66)	3.97 (1.11, 14.23)	**0.034**	2.16 (0.53, 8.82)	0.285
Employed/Business	19	3 (15.79)	Reference		Reference	
**Bed net use**						
No	6	5 (88.33)	8.33 (0.95, 73.13)	0.056	12.88 (1.27, 130.15)	**0.030**
Yes	152	57 (37.50)	Reference		Reference	
**SP receipt**						
<3 doses	158	64 (40.51)	-	-	-	-
≥3 doses	4	0 (0.00)				

HC = health centre; OR = odds ratio, PD = parasite density, 95% CI = 95% confidence interval, SP = sulphadoxine-pyrimethamine; Significant p-values are presented in bold.

### Factors influencing the prevalence of malaria in pregnancy using RDT

Factors associated with prevalence of malaria in pregnancy by RDT are summarized in Table 4. Bivariate logistic regression analysis revealed that receipt of three or more doses of IPTp-SP was associated with negative RDT. Maternal age younger than 20 years (OR = 1.87, *p* = 0.020), women from peri-urban area (OR = 2.59, *p* <0.001), paucigravidae (OR = 2.06, *p* = 0.005), women with low level of education (OR = 4.79, *p* = 0.003), peasants (OR = 3.04, *p* = 0.021) were associated with increased odds of malaria in pregnancy using RDT in crude analysis. In multivariable analysis while controlling for possible confounders, women delivering at Katoro rural health centre (AOR = 2.86, *p* <0.001) and those with low level of education (AOR = 3.51, *p* = 0.023) remained significantly associated with malaria in pregnancy based on RDT.

**Table 4:** **Analysis of factors associated with malaria in pregnancy by rapid diagnostic test among delivering women**

Variable	MIP by RDT		Crude OR	P-value	Adjusted OR	P-value
	N	No. RDT-positive (%)	(95% CI)		(95% CI)	
**Maternal age**						
<20 years	97	27 (27.84)	1.87 (1.11, 3.18)	**0.020**	1.05 (0.54, 2.05)	0.888
≥20 years	334	57 (17.07)	Reference		Reference	
**Health facility**						
Geita hospital	265	36 (13.58)	Reference		Reference	
Katoro HC	166	48 (28.92)	2.59 (1.59, 4.21)	**<0.001**	2.86 (1.69, 4.86)	**<0.001**
**Gravidity**						
Paucigravidae	227	56 (24.67)	2.06 (1.25, 3.39)	**0.005**	2.37 (1.29, 4.38)	**0.006**
Multigravidae	204	28 (13.73)	Reference		Reference	
**Level of education**						
None/Primary	360	80 (22.22)	4.79 (1.69, 13.53)	**0.003**	3.51 (1.19, 10.39)	**0.023**
Secondary/Higher	71	4 (5.63)	Reference		Reference	
**Occupation**						
Peasant/livestock	370	79 (21.35)	3.04 (1.18, 7.85)	**0.021**	1.86 (0.68, 5.10)	0.230
Employed/Business	61	5 (8.20)	Reference		Reference	
**Bed net use**						
No	10	4 (40.00)	Reference		Reference	
Yes	403	77 (19.11)	0.35 (0.10, 1.28)	0.115	0.28 (0.07, 1.10)	0.067
**SP receipt**						
<3 doses	405	84 (20.74)	-	-	-	-
≥3 doses	26	0 (0.00)				

HC = health centre; RDT = rapid diagnostic test, MIP = malaria in pregnancy, OR = odds ratio, 95% CI = 95% confidence interval, SP = sulphadoxine-pyrimethamine; Significant p-values are presented in bold.

### Uptake of IPTp-SP and peripheral malaria by microscopy at delivery

The association between IPTp-SP use and peripheral malaria by microscopy adjusted by other covariates is summarized in Table 5. Having employment or being a business woman, secondary or higher level of education attainment, receipt of three or more doses of IPTp-SP, attendance of four or more ANC visits and bed net use were associated with decreased trend towards odds of peripheral malaria in bivariate analysis, although these variables did not reach statistical significance. Only women delivering at Katoro rural health centre had a significant trend towards increased odds of peripheral malaria by microscopy in both bivariate (OR = 1.72, *p* = 0.012) and multivariable analysis (AOR = 2.77, *p* = 0.014).

**Table 5:** **Analysis of factors associated with peripheral malaria by microscopy among delivering women**

Variable	Peripheral malaria		Crude OR	P-value	Adjusted OR	P-value
	N	No. peripheral malaria positive (%)	(95% CI)		(95% CI)	
**Maternal age**						
<20 years	97	33 (34.02)	130 (0.80, 2.10)	0.291	1.28 (0.76, 2.14)	0.350
≥20 years	334	95 (28.44)	Reference		Reference	
**Health facility**						
Geita hospital	265	67 (25.28)	Reference		Reference	
Katoro HC	166	61 (36.75)	1.72 (1.13, 2.61)	**0.012**	1.76 (1.12, 2.77)	**0.014**
**Level of education**						
None/Primary	360	113 (31.39)	1.71 (0.93, 3.15)	0.086	1.29 (0.66, 2.53)	0.458
Secondary/Higher	71	15 (21.13)	Reference		Reference	
**Occupation**						
Peasant/livestock	370	116 (31.35)	1.86 (0.96, 3.64)	0.068	1.43 (0.69, 2.98)	0.340
Employed/business	61	12 (19.67)	Reference		Reference	
**SP receipt**						
<3 doses	405	124 (30.62)	Reference		Reference	
≥3 doses	26	4 (15.38)	0.41 (0.14, 1.22)	0.110	0.68 (0.22, 2.14)	0.489
**ANC visits**						
1-3	276	88 (31.88)	1.35 (0.87, 2.09)	0.186	1.32 (0.83, 2.12)	0.243
≥4	155	40 (25.81)	Reference		Reference	
**Bed net use**						
No	10	5 (50.00)	Reference		Reference	
Yes	403	118 (29.28)	0.41 (0.12, 1.45)	0.169	0.39 (0.12, 1.39)	0.164

ANC = antenatal care; OR = odds ratio, 95% CI = 95% confidence interval, SP = sulphadoxine-pyrimethamine; Significant p-values are presented in bold.

### Uptake of IPTp-SP and placental parasitaemia by microscopy

**Figure 1:**
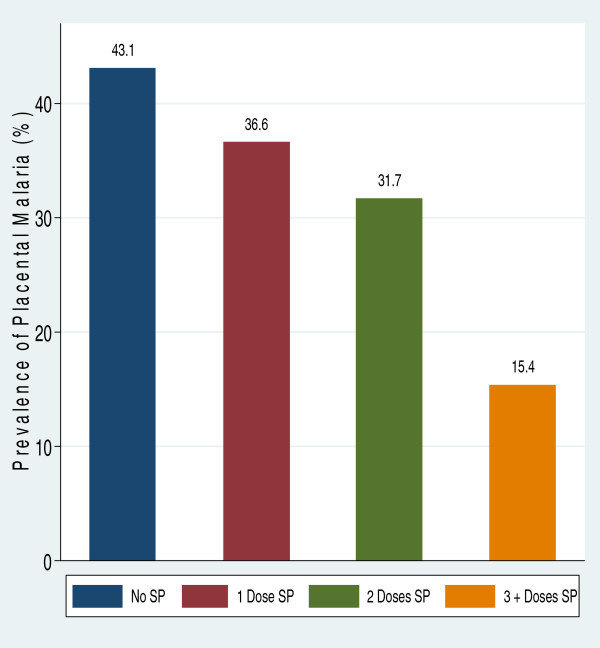
**Prevalence of placental parasitaemia by frequency of sulphadoxine-pyrimethamine doses among 431 delivering women.** The prevalence of placental parasitaemia was lowest among women who received three or more doses of SP during pregnancy and was highest among expectant women who did not receive any dose.

As shown in Figure 1, the prevalence of placental malaria was lowest (15.38%) among women who received three or more IPTp-SP doses and was highest (43.1%) among those who did not receive any dose of SP. Placental malaria parasitaemia was higher among women aged below 20 years (*p* = 0.188) and among paucigravidae (*p* = 0.084) in bivariate analysis although the difference was not statistically significant. In multivariable analysis (Table 6), adjusted for age, health facility, gravidity, bed net use and ethnicity; women who took three or more doses of IPTp-SP had a lower odds of placental parasitaemia when compared with those who took less than doses (AOR = 0.31, *p* = 0.039).

**Table 6:** **Analysis of factors associated with placental malaria by microscopy among delivering women**

Variable	Placental malaria (PM)		Crude OR	P-value	Adjusted OR	P-value
	N	No. PM positive (%)	(95% CI)		(95% CI)	
**Maternal Age**						
<20 years	97	42 (43.30)	1.36 (0.86, 2.16)	0.188	1.08 (0.62, 1.89	0.790
≥20 years	334	120 (35.93)	Reference		Reference	
**Ethnicity**						
Sukuma/Zinza	309	122 (39.48)	1.34 (0.86, 2.08)	0.197	1.18 (0.74, 1.89)	0.489
Other Tribes	122	40 (32.79	Reference		Reference	
**Health facility**						
Geita hospital	265	93 (35.09)	Reference		Reference	
Katoro HC	166	69 (41.57)	1.32 (0.88, 1.96)	0.177	1.29 (0.84, 1.98)	0.239
**Gravidity**						
Paucigravidae	227	94 (41.41)	1.41 (0.95, 2.09)	0.084	1.43 (0.89, 2.30)	0.135
Multigravidae	204	68 (33.33)	Reference		Reference	
**SP receipt**						
<3 doses	405	158 (39.01)	Reference		Reference	
≥3 doses	26	4 (15.38)	0.28 (0.10, 0.84)	**0.023**	0.31 (0.10, 0.94)	**0.039**
**Bed net use**						
No	10	6 (60.00)	2.48 (0.69, 8.92)	0.165	2.53 (0.69, 9.26)	0.159
Yes	403	152 (37.72)	Reference		Reference	

HC = health centre; OR = odds ratio, 95% CI = 95% confidence interval, PM = placental malaria; SP = sulphadoxine-pyrimethamine; Significant p-values are presented in bold.

### The influence of placental malaria parasitaemia and other covariates on pre-term delivery

Factors associated with pre-term delivery are shown in Table 7. In bivariate analysis, the odds of delivering a pre-term infant was higher among: 1) young women (*p* = 0.008); 2) delivering at Katoro rural health centre (*p* = 0.002); 3) women with positive placental smear compared to uninfected (*p* = 0.001) and taking the last dose of IPTp-SP more than four weeks before delivery (*p* = 0.069). In multivariable analysis, being of young age <20 years (*p* = 0.029), having placental malaria (*p* = 0.002), and taking the last dose of SP more than four weeks before delivery (*p* = 0.026) remained significantly associated with increased odds of pre-term delivery.

**Table 7:** **Secondary analysis of factors associated with pre-term delivery among delivering women**

Variable	Pre-term delivery^§^		Crude OR	P-value	Adjusted OR	P-value
	N	No. PTD (%)	(95% CI)		(95% CI)	
**Maternal age**						
<20 years	97	21 (21.65)	2.22 (1.23, 4.01)	**0.008**	2.92 (1.12, 7.65)	**0.029**
≥20 years	334	37 (11.08)	Reference		Reference	
**Health facility**						
Geita hospital	265	25 (9.43)	Reference		Reference	
Katoro HC	166	33 (19.88)	2.38 (1.36, 4.18)	**0.002**	2.02 (0.65, 6.35)	0.227
**Peripheral malaria**						
Positive	128	19 (14.84)	1.18 (0.65, 2.13)	0.584	0.36 (0.11, 1.10)	0.073
Negative	303	39 (12.87)	Reference		Reference	
**Level of education**						
None/Primary	360	53 (14.72)	2.28 (0.88, 5.92)	0.091	1.69 (0.45, 6.33)	0.439
Secondary/Higher	71	5 (7.04)	Reference		Reference	
**Placental malaria**						
Positive	162	33 (20.37)	2.50 (1.42, 4.38)	**0.001**	4.67 (1.74, 12.58)	**0.002**
Negative	269	25 (9.25)	Reference		Reference	
**MIP by RDT**						
Positive	84	16 (19.05)	1.71 (0.91, 3.22)	0.097	2.13 (0.69, 6.59)	0.188
Negative	347	42 (12.10)	Reference		Reference	
**SP receipt**						
<3 doses	405	55 (13.58)	Reference		Reference	
≥3 doses	26	3 (11.54)	0.83 (0.24, 2.86)	0.768	0.55 (0.13, 2.33)	0.416
**Last SP time lapse**						
≤4 weeks	26	14 (53.85)	0.47 (0.21, 1.06)	0.069	0.38 (0.15, 0.97)	**0.042**
>4 weeks	238	170 (71.43)	Reference		Reference	

HC = health centre; RDT = rapid diagnostic test, MIP = malaria in pregnancy, PTD = pre-term delivery, § = Assessed by first day of last menstrual period or by New Ballard Score; Significant p-values are presented in bold.

### The influence of placental malaria parasitaemia and other covariates on infant birth weights

The association between placental malaria and infant birth weight is summarized in Table 8. Mean birth weights were significantly lower: in infants born by mothers younger than 20 years (2930 ± 414 g) compared to mothers aged 20 years or older (3271 ± 436 g) (*p* <0.001), by paucigravidae (3141 ± 460 g) compared to multigravida (3289 ± 436 g) (*p* <0.001), by women with less than four ANC visits (3141 ± 460 g) compared to four or more visits (3289 ± 428 g), (*p* = 0.001), by women with placental parasitaemia (3096 ± 448 g) than that of uninfected counterparts (3254 ± 448 g) (*p* <0.001), and preterm birth (2728 ± 503 g) than that of term delivery (3267 ± 400 g), (*p* <0.001). On multiple linear regression analysis (Table 8), maternal age younger than 20 years (*p* <0.001), paucigravidae (*p* = 0.002), women with placental malaria (*p* = 0.039), preterm delivery (*p* < 0.001) and ANC visits less than four (*p* = 0.001) remained statistically significantly associated with delivery of infants with lower mean birth weights. The mean birth weights of infants born by women who received three or more doses of IPTp-SP were higher (3311 ± 476 g) than those who received less than three doses (3187 ± 452 g); although this variable did not reach statistical significance (*p* = 0.713).

**Table 8:** **Secondary analysis of factors associated with low birth weight delivery among delivering women**

Variable	Mean birth weight (SD) g	Coefficient	P-value	Coefficient	P-value
		(95% CI)		(95% CI)	
**Maternal age**					
<20 years	2,930 (414)	**−341** (−439, −243)	**<0.001**	−193 (−297, −90)	**< 0.001**
≥20 years	3,271 (436)	Reference		Reference	
**Gravidity**					
Paucigravidae	3,141 (460)	−243 (−326, −160)	**<0.001**	−134 (−220, −48)	**0.002**
Multigravidae	3,289 (428)	Reference		Reference	
**ANC visits**					
1-3 visits	3,141 (460)	−148 (−237, −60)	**0.001**	−128 (−206, −50)	**0.001**
≥4 visits	3,289 (428)	Reference		1	
**Placental malaria**					
Positive	3,096 (448)	−158 (−245, −70)	**<0.001**	−82 (−160, −4)	**0.039**
Negative	3,254 (448)	Reference		1	
**SP receipt**					
<3 doses	3,187 (452)	−125 (−305, 55)	0.174	30 (−129, 188)	0.713
≥3 doses	3,311 (476)	Reference		Reference	
**Gestational age**					
Pre-term delivery	2,728 (503)	**−539 (−654, −424)**	**<0.001**	**−**479 (−589, −369)	**<0.001**
Term delivery	3,267 (400)	Reference		Reference	

ANC = antenatal care; SD = standard deviation, 95%CI = 95% confidence interval, Significant p-values are presented in bold.

## Discussion

### Uptake of IPTp-SP

In SSA, the effectiveness of IPTp-SP in prevention of malaria-related adverse pregnancy outcomes is well documented [13–15, 21]. However, the coverage of recommended doses of IPTp-SP in SSA is still very low [16]. In the present study, the uptake of at least one and two doses of SP were consistent with national estimates from the 2011–12 Tanzania HIV/AIDS and Malaria Indicator Survey report of 60% and 32%, respectively [18]. However, the uptake of at least two doses of SP observed in the present study was lower than 35% reported previously in the same study area [28], reflecting the inconsistency of drug supply at the time of survey in the given area. The uptake of the recommended three doses of IPTp-SP has never been evaluated in Tanzania. The uptake of at least three doses of IPTp-SP in the present study was very low. The uptake of IPTp-SP in the present study is far below the Roll Back Malaria (RBM) target for all pregnant women living in areas with stable transmission in SSA to receive IPTp-SP by the end of 2015 [29].

Receipt of ≥ three doses of IPTp-SP in the present study was higher among women making ≥ four ANC clinic visits compared to women making fewer visits and among women residing in rural areas. These findings are consistent with that of Exavery *et al.* conducted in six selected districts of Tanzania [28], in Ghana by Hommerich *et al.*[15], in rural Burkina Faso by Gies *et al.*[14], and in Mali by Hill *et al.*[30]. ANC attendance is the entry point for receiving IPTp-SP; the more visits the woman attends the higher the number of SP she will receive. The ANC attendance of at least four visits was 36% but the uptake of at least three doses of SP during pregnancy was only 6% in this study, suggesting missed opportunities when women attended clinics but were not given IPTp-SP. The high ANC attendance rate with low uptake of IPTp can be explained in part by periodic shortage of SP in the health facility, poor adherence by health providers to provision of IPTp-SP and women’s negative attitudes towards the use the drug during pregnancy [30, 31].

### Prevalence of placental malaria

The prevalence of malaria infection using RDT, peripheral and placental smears was 19.49%, 29.70% and 37.60% respectively. The sensitivity of RDT over microscopy in diagnosis of malaria in pregnancy was very low (<40%). The difference can be explained, in part, that the performance of RDT depends on the level parasitaemia and the tests are more sensitive when parasite densities >100 parasites/μL [32]. In the present study, over half of women with asymptomatic parasitaemia had low parasite density <100 parasites/μL. The finding is similar to an observational study conducted in Nigeria whereby SD Bioline RDT sensitivity was reported to be 47% among asymptomatic children [33] but contrary to a report from Burkina Faso where RDT had sensitivity of 89% [34]. The prevalence of malaria in pregnancy by RDTs was consistent with a recent report from Zambia by Tan *et al.* where 19.77% [35]*Plasmodium falciparum* infection was observed in 94.05% of delivering women as either mixed infection (32.14%) or as a mono-infection (61.91%). This is consistent with previous reports that falciparum malaria was the predominant infection observed among pregnant women [3, 9]. The prevalence of placental malaria by thick film smear was high (37.6%) in this study. This prevalence of placental malaria can be attributed, in part, to increased transmission intensity with elevated re-infection rates between the last dose of SP and delivery, low uptake of IPTp-SP and that the study was conducted during the end of rainy season where transmission is at its highest peak [14]. The prevalence of placental malaria by microscopy is similar to those reported in Cameroon (33.7%) and Ghana (38.6%) [36, 37] but was higher than previously reported in Uganda (17.5%) and among Cameroonian parturient women (25.5%) [38, 39]. However, the prevalence of placental malaria in this study is lower than that reported by Ezebialu *et al.* (63.3%) and Aribodor *et al*. (64.4%) in Nigeria respectively [5, 40].The differences in the prevalence of placental malaria across the regions can be explained by geographical variations in malaria transmission intensities, seasonality, differences in the characteristics of study participants, coverage of preventive measures, and study designs.

### IPTp-SP use and malaria in pregnancy

In Geita district, uptake of three or more doses of IPTp-SP was a perfect predictor of negative malaria in pregnancy using RDT in bivariate analysis. Moreover, receipt of three or more doses of SP was associated with significantly reduction of placental parasitaemia, which supports the recent policy change from two to three or more doses of IPTp-SP in Tanzania. The superiority of three or more over less than three doses of SP in reduction of placental malaria and birth outcomes is consistent with a recent clinical trial conducted in Mali [13]. The significant protection of SP against placental malaria is not surprising in an area with moderate to high SP *P. falciparum* resistance [41].This supports the WHO recommendation to continue use of SP as IPTp even in areas with high levels of SP resistance [42]. In north-eastern Tanzania where there is high SP resistance, Harrington *et al.* and Minja *et al.* reported loss of efficacy of SP as IPTp and exacerbation of adverse birth outcomes [43, 44]. However, receipt of ≥ three doses of IPTp-SP was not associated with reduced odds of peripheral malaria in the present study. The lack of association could be explained, in part, by the fact that a small proportion of women took the recommended three or more doses of SP during pregnancy and that this may have reduced the power to produce an effect.

### Placental malaria and pre-term delivery

The number of doses of IPTp-SP taken was not associated with reduced risk of pre-term delivery. However, women who received the last dose of IPTp-SP within four weeks of delivery had a 62% odds reduction of delivering pre-term infants. This is not surprising as the protection period of IPTp-SP is around four weeks.

The presence of placental parasitaemia was associated with a significant trend towards increased odds of pre-term delivery. This finding concurs with those reported previously across SSA where malaria is highly endemic [13, 45]. In contrast, in Cameroon, placental parasitaemia was not associated with pre-term delivery [46]. The pathophysiology of placental parasitaemia and pre-term delivery is uncertain. However, malaria-infected placentas often release inflammatory immune responses, such as antibodies, cytokines and macrophages, which may be responsible for early uterine stimulation initiating labour [3]. These mechanisms are thought to result in pre-term labour.

### Placental malaria and infant birth weight

The prevalence of LBW in this study was very low (2.32%) and the overall mean birth weight was high (3194.20 ± 2187 g). The uptake of three or more doses of SP was not associated with a significant increase in mean birth weight of new-borns. The lack of association in this study could be explained, in part, by the low uptake of optimal doses of SP and high mean birth weight. In addition, the small sample size of the current study may not provide the power needed to detect the effect on birth weight.

Infants born to women who attended ANC clinics four or more times were on average 128 g heavier than infants of women who had attended ANC clinics less than three times. The association between ANC clinic visits and mean birth weight can be explained by the fact that more visits are associated with the receipt of more SP when readily available, and women supplemented with haematinics, counselled on nutrition during pregnancy, all of which are associated with improved birth weight. Moreover, infants born to young mothers and those born prematurely were 193 g and 128 g lighter than those born to older mothers and those born at term. Delivery of lower birth weight infants among young mothers can be explained by the fact that this age group is susceptible to malaria infection owing to inadequate pregnancy-associated immunity. For example, anticytoadherent antibodies that inhibit adhesion of *P. falciparum* to chondroitin sulphate A (CSA) is under-developed among first-time young pregnant women, compared to older non-first-time mothers with adequate immunity as a result of repeated exposure to malaria parasites [46]. Placental malaria was not associated with delivery of LBW owing to low prevalence of LBW in the current study that could not provide sufficient power to detect any potential associations. The low prevalence of LBW despite high prevalence of placental malaria can be explained by fact that there could be other causes of intrauterine foetal growth such as maternal malnutrition, maternal/foetal constitutional factors and other environmental non-infectious factors [47]. However, analysing birth weights as continuous variable, women with placental parasitaemia were more likely to deliver lower birth weight infants than uninfected women. The association between placental parasitaemia and low mean birth weight in this study is consistent with previous reports [3, 5, 9, 45, 48]. The delivery of LBW can be caused by pre-term delivery or by placental malaria sequestration causing intrauterine growth restriction (IUGR) [9, 48]. Foetal growth restriction as a result of placental malaria sequestration is mediated by accumulation of infected erythrocytes into the maternal placental intervillous spaces that trigger chronic inflammatory responses leading to placental insufficiency [2, 48].

### Limitations

This study was an analytical, cross-sectional study. Although efforts were made to control for confounders at the design and analyses stages, unidentified confounders may still have affected the observations. In addition, it is possible that not the entire dose of SP given to women was actually taken, as the receipt of SP was self-reported and some women did not consume SP as a directly observed therapy. There may have been a possibility of over-estimating IPTp-SP use in the study, as some participants may have tried to please the interviewer. A significant number of women who deliver outside health facilities in Tanzania may have had more adverse birth outcomes. However, this study had the limitation of recruiting study participants at delivery units. Therefore, this study was not representative of the wider population in north-western Tanzania. The lack of association between the uptake of > three doses of IPTp-SP and birth outcomes (pre-term delivery and LBW) could be attributed to the small sample size and the proportion of women who took the recommended dose.

## Conclusions

Placental parasitaemia is common in Geita district and is likely to be associated with pre-term delivery. Uptake of IPTp with SP is very low among pregnant women attending ANC clinics in public health facilities of Geita district. However, the uptake of three or more doses of IPTp-SP in this area with highly intense malaria transmission is associated with reduction of placental malaria. Effort is needed to scale-up the uptake of optimal doses of IPTp-SP especially in peri-urban areas of Geita district. A longitudinal study may be required to evaluate the relationship between the uptake of recommended doses of IPTp-SP and birth outcomes in this area. Given substantial utilization of ANC in contrast to the low uptake of IPTp-SP in this population, qualitative research studies are needed to determine any underlying barriers of utilization of intermittent preventive treatment of malaria with SP during pregnancy.

## Abbreviations

ANC: Antenatal care
AOR: Adjusted odds ratio
ARC: Adjusted Regression Coefficient
BMC: Bugando Medical Centre
CI: Confidence interval
CSA: Chondroitin surface A
CUHAS: Catholic University Health of Allied Sciences
EDTA: Ethylene diamine tetra acetic acid
GMPD: Geometric mean parasite density
HIV: Human immunodeficiency virus
HRP II: Histidine-rich protein II
IPTp: Intermittent preventive treatment of malaria in pregnancy
IPTp-SP: Intermittent preventive treatment of malaria in pregnancy with sulphadoxine-pyrimethamine
IUGR: Intrauterine growth restriction
LBW: Low birth weight
RDT: Rapid diagnostic test
*Pfdhfr*: *Plasmodium falciparum dihydrofolate reductase*
*Pfdhps*: *Plasmodium falciparum dihydropteroate synthase*
pLDH: Parasite lactase dehydrogenase
RBM: Roll Back Malaria
SSA: Sub-Saharan Africa
SP: Sulphadoxine-pyrimethamine
WHO: World Health Organization.
